# Perioperative management protocol for abdominal wall hernia repair in a public reference healthcare unit in Amazonas - Brazil

**DOI:** 10.1590/0100-6991e-20253820-en

**Published:** 2025-09-23

**Authors:** THIAGO GUIMARÃES MATTOS DE SOUZA, SILVANIA DA CONCEIÇÃO FURTADO, MARIA CAROLINA COUTINHO XAVIER SOARES, FERNANDO LUIZ WESTPHAL

**Affiliations:** 1 - Universidade Federal do Amazonas - UFAM, Programa de pós-graduação em cirurgia - PPGRACI - Manaus - AM - Brasil; 2 - Universidade Estadual do Amazonas - UEA - Manaus - AM - Brasil

**Keywords:** Enhanced Recovery After Surgery, Perioperative Care, Combined Modality Therapy, Hernia, Abdominal, Hernia, Ventral, Hérnia Abdominal, Hérnia Ventral, Recuperação Pós-cirúrgica melhorada, Assistência Perioperatória, Terapia Combinada

## Abstract

**Introduction:**

Abdominal hernias are frequent surgical conditions that incur high hospital costs and significantly affect patients socioeconomic lives. It is crucial to reassess and update operational protocols, as several aspects of perioperative care require revision, especially following the introduction of protocols aimed at accelerating postoperative recovery.

**Objective:**

To develop a protocol for perioperative procedures in abdominal wall hernia repair surgeries.

**Method:**

This is a prospective study with a mixed approach, utilizing a modified Delphi consensus method. The sample comprised physicians with residency in general surgery, with a minimum of five years of experience and routine involvement in treating abdominal wall hernias. Questionnaires were administered in three stages, addressing various aspects related to perioperative care in patients undergoing abdominal wall hernia repair surgeries. For the analysis of collected data, the Content Validity Index (CVI) was calculated based on the percentage of agreement among participants, and Cronbachs Alpha coefficient was used to assess the internal consistency of the questionnaire.

**Results:**

The study generated a protocol for perioperative clinical-surgical procedures focused on abdominal wall hernia repair surgeries.

**Conclusion:**

The development of a consensus among specialists resulted in the creation and publication of a protocol for perioperative clinical-surgical procedures in abdominal hernia repair surgeries.

## RESUMO

**Objetivo:**

As hérnias abdominais são condições cirúrgicas frequentes que acarretam elevados custos hospitalares e afetam significativamente a vida socioeconômica dos pacientes. É importante reavaliar e atualizar os protocolos operacionais, dado que vários aspectos dos cuidados perioperatórios necessitam de revisão, especialmente após a introdução de protocolos que visam acelerar a recuperação pós-operatória.

**Objetivo:**

Elaboração de um protocolo de procedimentos perioperatórios para cirurgias de correção de hérnias da parede abdominal.

**Método:**

Trata-se de um estudo prospectivo de abordagem mista, utilizando o método Delphi de consenso modificado. A amostra foi composta por médicos com residência em cirurgia geral, com no mínimo cinco anos de experiência e atuação rotineira no tratamento de hérnias da parede abdominal. Os questionários foram aplicados em três etapas, abordando variados aspectos relacionados aos cuidados perioperatórios em pacientes submetidos a cirurgias de correção de hérnias da parede abdominal. Para a análise dos dados coletados, foram calculados o Índice de Validação de Conteúdo (IVC) baseando-se na porcentagem de concordância entre os participantes, e o coeficiente Alfa de Cronbach, para avaliar a consistência interna do questionário.

**Resultados:**

O estudo gerou um protocolo de condutas clínico-cirúrgicas perioperatórias voltado para cirurgias de correção de hérnias da parede abdominal.

**Conclusão:**

A elaboração de um consenso entre especialistas resultou na criação e publicação de um protocolo para condutas clínico-cirúrgicas perioperatórias em cirurgias de correção de hérnias abdominais.

## INTRODUCTION

Correction of abdominal hernias is among the most frequent surgical interventions on a global scale^1^ ^,^ ^2^. It is estimated that 20 million hernioplasties are performed annually in the world. In the United States alone, more than one million abdominal wall hernias are repaired each year, most of which consist of inguinal hernias^3^ ^,^ ^4^. 

A survey conducted via DataSUS, in February 2025, on abdominal hernia surgeries performed in Brazil by the Unified Health System (SUS), revealed that this condition affects between 20% and 25% of Brazilian adults. Inguinal hernias represent 75% of cases and are the main cause of surgical interventions, with 323,045 procedures performed^5^.

Perioperative practices, from preparation to recovery, are determinant of surgical success^6^. Hernioplasty can generate high costs and impact patients due to sick leave and risk of recurrence^7^. The treatment of abdominal hernias should consider the surgeon’s experience, patient characteristics, and available resources^8^ ^-^ ^10^. Laparoscopic techniques, when feasible, are preferred due to the shorter recovery time and lower risk of chronic pain, with the use of mesh being the first option^10^. Laparoscopy reduces postoperative pain and the risk of infection but can cause hernias at the trocar insertion sites and prolong surgical time^8^ ^,^ ^11^ ^,^ ^12^. The Lichtenstein technique can reduce operative time and recurrence rate^12^. In clinical practice, inconsistent and non-evidence-based approaches may cause complications^13^ ^-^ ^16^.

Perioperative practices, often empirical, become obsolete with scientific developments^17^ ^,^ ^18^. Modernizing operational protocols is essential, especially with the emergence of programs such as Enhanced Recovery After Surgery (ERAS) and ACERTO, which aim to accelerate recovery^15^ ^,^ ^17^ ^,^ ^19^.

The concept of “improving post-surgical recovery”, introduced in Denmark in the 1990s by Dr. Henrik Kehlet, integrates interventions to improve patient safety and recovery^20^ ^,^ ^21^. Recovery in abdominal surgeries is influenced by perioperative approaches recommended by international protocols, such as the International Guidelines for Groin Hernia Management, although regional adaptations are necessary^9^ ^,^ ^10^. 

In this sense, the objective of this study was to identify the perioperative procedures performed in a public health unit located in Northern Brazil to accelerate abdominal wall surgeries’ postoperative recovery. Based on the results obtained, we proposed the creation of a protocol of perioperative conducts in abdominal wall hernia surgeries. 

## METHODS

This is a prospective study with a mixed approach aimed at the production, analysis, and improvement of a consensus instrument using the Delphi method^22^. We used the AGREE II checklist to guide the elaboration of the Protocol^23^. The study was approved by the Ethics in Research Committee of the Federal University of Amazonas (CEP-UFAM), under the number CAAE 76727223.7.0000.5020. 

We conducted the study in three stages, using electronic questionnaires. We stratified the sample by convenience, opting for the formation of a panel of 32 specialist surgeons with medical residency in general surgery, trained in the management of surgical conditions, who routinely follow-up their surgical patients, from preoperative period to surgical discharge^24^.

The inquiries contained in the questionnaires addressed aspects of the perioperative care of patients undergoing abdominal wall hernioplasty. The variables discussed in the questionnaires were:

- Preoperative clinical evaluation of the patient.
- Evaluation of the preferred surgical access route (laparotomy or laparoscopy).
- Investigation and management of comorbidities and addictions during the pre and postoperative period.
- Preoperative nutritional assessment.
- Evaluation of the pre and postoperative fasting time.
- Evaluation of the need and volume of hydration in the postoperative period.
- Assessment of the need for preoperative multidisciplinary prehabilitation
- Evaluation of the patient’s mobilization time in the postoperative period.
- Indications and management of antibiotic prophylaxis.
- Indications for pre- and postoperative immunonutrition.
- Need to use drains and catheters.
- Assessment and management of the risk of deep vein thrombosis pre- and postoperatively.
- Assessment of the need to control pain, nausea, and vomiting in the postoperative period.

We created the questionnaires on the Google Forms^®^ platform and sent then individually to the participants via email. The estimated time for filling out was about one hour, with a suggested deadline of 15 days for return. 

### Procedures

Stage 1 - Formulation and Application of the First Questionnaire: The first questionnaire was elaborated based on scientific information identified in an integrative literature review and prioritized questions in which each professional could express their ideas for the delimitation of the topics and themes addressed^25^ ^-^ ^27^. We presented 39 questions, of which 34 referred to perioperative approaches aimed at the treatment of abdominal wall hernias, and five covered participants socioeconomic profile. We submitted the answers to the agreement rate and to the Chi-square test of adherence to calculate the p-value.

Stage 2 - Formulation and Application of the Second Questionnaire: After the evaluation of the questions derived from the initial questionnaire, was developed a second one. This consisted of 27 texts formulated from the answers that obtained greater agreement between the questions answered by the participants in the first questionnaire, which was later submitted to the experts’ evaluation. To evaluate the answers, w adopted a four-point Likert scale after each text and calculated the Cronbach’s Alpha to assess the internal consistency of the questionnaire, thus ensuring the quality and reliability of the data collected, as well as the percentage of the agreement rate^28^ ^-^ ^30^.

Stage 3 - Elaboration of the Consensus Proposal: In this stage, we prepared a consensus proposal by calculating the approval rate of the protocol presented, using the 4-point Likert scale. In this phase, we calculated the Content Validation Index (CVI) for content validation in the preoperative, intraoperative, and postoperative periods^31^ ^,^ ^32^.

## RESULTS

The socio-professional profile of the 32 specialists participating in the study is described in Table 1. 

**Table 1:** Socio-professional profile of the research participants.

Profile of the participants		N	%
Sex	Female	4	12.5%
	Male	28	87.5%
Age group (years)	30 to 35	2	6.3%
	36 to 40	6	18.8%
	41 to 45	11	34.4%
	46 to 50	8	25.0%
	>50	5	15.6%
Time of medical training (years)	5 to 10	3	9.4%
	11 to 20	18	56.3%
	>20	11	34.4%
Time to end of Medical Residency (years)	<10	5	15.6%
	10 to 19	20	62.5%
	≥20	7	21.9%
Degree of education	Specialization	13	40.6%
	Subspecialization	24	75.0%
	Masters	5	15.6%
	Doctorate	1	3.1%

Source: Authors

Most of the participants who answered the first questionnaire were male, representing 28 individuals (87.5%). As for the age group, it ranged from 30 to more than 50 years, the highest percentage being between 41 and 45 years (34.4%). The time since medical graduation ranged from five to more than 20 years, with the highest percentage between 11 and 20 years (56.3%). Specialists with a degree between 10 and 19 years made up the largest portion of the sample (62.5%). The others were distributed as follows: <10 years (15.6%), >20 years (21.9%). As for the level of training of the 32 surgeons participating in the study, 24 (75%) had, in addition to residency in general surgery, another surgical specialty such as vascular surgery, digestive system, head and neck, etc. Regarding the other participants, 13 (40.6%) had only general surgery residency. Five (15.6%) had a master’s degree and one (3.1%) had a doctorate (PhD), in addition to a residency in general surgery.

We divided the 34 questions according to affinity with the results and evaluated them in three categories: the first 13 questions related to the preoperative period, nine, to the intraoperative one, and 10, postoperative (Tables 2, 3 and 4). 

**Table 2:** Agreement of answers of the first phase of the questionnaire (Preoperative).

Phase 1: Preoperative	Agreement between participants		p-value
Question 01 - Evaluation of alcohol intake, smoking, hypertension, diabetes, HIV infection and viral hepatitis	28	80.0%	0.0004*
Question 02 - Smoking cessation for an adequate period before surgery	23	65.7%	0.0630
Question 03 - Timely cessation of alcohol use prior to surgery	22	62.9%	0.0630
Question 04 - Referral to physical prehabilitation programs before surgery	30	85.7%	<0.0001*
Question 05 - Discussion and provision of preoperative guidance in addition to the informed consent form	22	62.9%	0.1282
Question 06 - Routine preoperative nutritional screening	27	77.1%	0.0013*
Question 07 - Nutritional supplementation in malnourished patients	33	94.3%	<0.0001*
Question 08 - Referral to a specialist for preoperative nutritional intervention	25	71.4%	0.0112*
Question 09 - Prescription of immunonutrition	24	68.6%	0.0280*
Question 10 - Start of preoperative immunonutrition in advance before surgery	28	80.0%	0.0004*
Question 11 - Average duration of preoperative fasting recommended for abdominal hernia surgeries	24	68.6%	0.0280*
Question 12 - Fast-absorbing carbohydrates in the preoperative period	24	68.6%	0.0280*
Question 13 - Fast-absorbing carbohydrates in the preoperative period in diabetic patients	19	54.3%	0.6121

Source: Authors

**Table 3:** Agreement of answers of the first phase of the questionnaire (Intraoperative).

Phase 1: Intraoperative	Agreement between participants		p-value
Question 14 - Regular antimicrobial prophylaxis in abdominal hernia repair	33	94.3%	<0.0001*
Question 15 - Maintenance of antibiotic prophylaxis after surgery	26	74.3%	0.0041*
Question 16 - Risk scoring systems to predict deep vein thrombosis	23	65.7%	0.0630
Question 17 - Prophylaxis for venous thromboembolism	18	51.4%	0.8658
Question 18 - Local anesthetics	19	54.3%	0.6121
Question 19 - Preferred surgical route for abdominal hernioplasties	26	74.3%	0.0041*
Question 20 - Average pneumoperitoneum pressure in laparoscopic surgeries	20	57.1%	0.3980
Question 21 - Preventive measures against perioperative hypothermia	21	60.0%	0.2367
Question 22 - Nasogastric tubes	33	94.3%	<0.0001*
Question 23 - Abdominal drains	19	54.3%	0.6121
Question 24 - Bladder catheter	33	94.3%	<0.0001*

Source: Authors

**Table 4:** Agreement of answers of the first phase of the questionnaire (Postoperative).

Phase 1: Postoperative	Agreement between participants		p-value
Question 25 - Multimodal analgesia	31	88.6%	<0.0001*
Question 26 - Beginning of postoperative mobilization	17	48.6%	0.8658
Question 27 - Beginning of postoperative feeding	25	71.4%	0.0112*
Phase 1: Postoperative	Agreement between participants		p-value
Question 28 - Recommended diet in the immediate postoperative period	23	65.7%	0.0630
Question 29 - Regular hydration in the immediate postoperative period	26	74.3%	0.0041*
Question 30 - Volume normally prescribed for intravenous hydration	25	71.4%	0.0112*
Question 31 - Maintenance time of intravenous hydration	21	60.0%	0.2367
Question 32 - Prevention or treatment of ileus	33	94.3%	<0.0001*
Question 33 - Risk scoring system to predict nausea and vomiting	35	100.0%	<0.0001*
Question 34 - Prevention or treatment of nausea and vomiting	35	100.0%	<0.0001*

Source: Authors

Stage 1: We used the answers with the highest agreement among participants, which we aggregated to create texts distributed on specific subjects, resulting in 27 texts.

Stage 2: After the elaboration of the texts, we divided them by affinity into preoperative (12 texts), intraoperative (nine texts) and postoperative (six texts) conducts (Table 5).

**Table 5:** Agreement of answers of the second phase of the questionnaire.

Consensus Phase 2	Score Received	% Approval	Cronbach's Alpha
Preoperative	1416	91.9%	0.930
Intraoperative	1308	93.4%	0.931
Postoperative	814	96.9%	0.982
Consensus	3538	93.6%	0.953

Source: Authors

**Graph 1:**
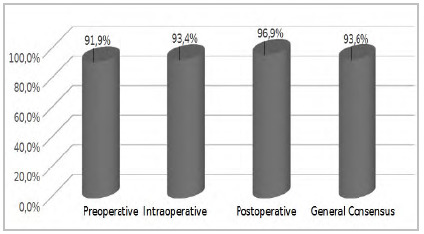
Preliminary consensus of answers of the second phase of the questionnaire.

Using the preliminary agreement answers (Graph 1) and the Cronbach’s Alpha Index with positive results in the evaluation of comprehension of the texts, we unified the data according to their degree of affinity in relation to the perioperative period (preoperative, intraoperative, and postoperative), resulting in the elaboration of three chapters for the Protocol. There was no need for the option of representing non-consensus questions, as all questions in the questionnaire were validated by more than 80% of the participants, without the need to be represented.

Step 3: During the preparation of the consensus concluding text, we identified the need for additional information. Such information referred to perioperative conducts. In view of this, we conducted a specific search in the literature to complement this relevant theme to the study.

**Graph 2:**
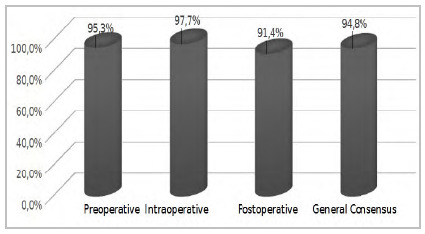
Preliminary Content Validation Index of answers for the third phase of the questionnaire.

The final draft of the consensus was then sent in protocol format to the 32 participants using the Likert scale at the end of each of the three chapters. The 32 participants answered the third stage of the study, without using the “Others” space, aimed at complementary comments (Table 6 and Graph 2).

**Table 6:** Agreement of answers of the third phase of the questionnaire.

Consensus Phase 3	Score Received	% Approval	CVI
Preoperative	122	95.3%	0.97
Intraoperative	125	97.7%	1.00
Postoperative	117	91.4%	0.88
Consensus	364	94.8%	0.95

Source: Authors; CVI: Content Validation Index.

At the end of the three stages, was developed a protocol of perioperative approaches for abdominal wall hernioplasties through a consensus of experts at a reference public health unit in Amazonas, Brazil. In the protocol, perioperative approaches were divided into preoperative, intraoperative, and postoperative approaches. The main points defined in each of the stages were:

### Preoperative Period:

1- Screening and Initial Assessment: Evaluate clinical conditions and lifestyle habits that may impact recovery, such as smoking, alcohol consumption, hypertension, diabetes, anemia, and serology for HIV and viral hepatitis. Screening helps determine the best time and type of surgical procedure.
2- Smoking Cessation: It is recommended to stop smoking for four to eight weeks before surgery to reduce respiratory and healing complications.
3- Alcohol Use: Abstaining from alcohol for at least four weeks prior to the procedure is advised to decrease the risk of postoperative infections.
4- Prehabilitation Skills: Implement multidisciplinary interventions to optimize the patient’s physical, nutritional, and mental conditions prior to surgery, especially in individuals with lower physiological reserve or malnourished.
5- Preoperative Education: Provide clear information about the surgical and anesthetic care plan to reduce patient anxiety and improve satisfaction.
6- Nutritional Screening: Perform a nutritional assessment, especially in medium and large surgeries, to identify and treat possible states of malnutrition.
7- Nutritional Supplementation: In cases of malnutrition, nutritional supplementation and, if applicable, immunonutrition with arginine and omega 3 is recommended to reduce the risk of surgical site infections.
8- Preoperative Fasting: Reduce the preoperative fasting period and consider the administration of fast-absorbing carbohydrates, such as maltodextrin, to decrease insulin resistance and improve postoperative recovery. The decision on the use of carbohydrates should consider the surgical size and the presence of diabetes

### Intraoperative Period:

1- Antibiotic Prophylaxis: The use of first-generation cephalosporins, such as cefazolin, is recommended without continuation after incision closure in clean or clean-contaminated wounds.
2- Deep Vein Thrombosis Prophylaxis: Use the Caprini score to assess the risk of deep vein thrombosis and determine the most appropriate prophylaxis, which may include intermittent pneumatic compression, elastic stockings, and/or heparin.
3- Perioperative Pain Management: Infiltration of local anesthetics, such as bupivacaine or lidocaine, into the surgical wounds is recommended to decrease postoperative pain.
4- Pneumoperitoneum Pressure Control: During laparoscopic surgeries, the recommended pneumoperitoneum should be kept below 12 mmHg, and not exceed 15 mmHg.
5- Prophylaxis of Hypothermia: In surgeries expected to last more than 30 minutes, measures to prevent intraoperative hypothermia should be taken, including the use of heated venous solutions and thermal blankets.
6- Probes and Drains:
  - Nasogastric tubes: They are not recommended routinely or prophylactically, and their use should be limited to specific situations.
  - Abdominal Drains: Its use is not recommended in rapid post-surgical recovery protocols, being indicated only according to the surgeon’s evaluation.
  - Foley urinary catheters: These should be removed as soon as possible, preferably within the first 24 hours after the procedure, to minimize the risk of urinary tract infection.

### Postoperative Period:

1- Multimodal analgesia: Use a postoperative multimodal analgesia approach, combining analgesics, nonsteroidal anti-inflammatory drugs, and local anesthetic blocks to reduce opioid dependence and facilitate recovery.
2- Early Mobilization: Encourage patient mobilization as early as possible, ideally within the first 12 hours after surgery, to prevent complications such as pneumonia, atelectasis, and venous thrombosis.
3- Early Refeeding: Initiate early oral feeding, with clear liquids in the immediate postoperative period, progressing to a complete liquid diet or solid feeding as the patient accepts, to promote the return of bowel function and reduce the length of hospital stay.
4- Postoperative Hydration: Prefer oral to intravenous hydration in the immediate postoperative period, limiting intravenous volume to 20-30ml/kg in 24 hours, and consider withholding intravenous hydration in the first 24 hours if clinically feasible.
5- Prevention and Treatment of Postoperative Ileus: Apply strategies such as early use of chewing gum, preference for less invasive surgeries, routine avoidance of nasogastric tubes, avoidance of excessive venous hydration, preference of non-opioid analgesics, early refeeding, and use of prokinetics.
6- Prevention of postoperative Nausea and Vomiting (PONV): Use the Apfel scale to assess the risk of PONV and apply adequate prophylaxis with antiemetics, considering the use of two or more antiemetic drugs for patients at high risk. Restriction or decrease in opioid use is also recommended to prevent PONV.

The final result was made available as a digital book, identified by ISBN 978-65-5866-420-8 and DOI 10.36229/978-65-5866-420-8 through the Link:

https://www.poisson.com.br/livros/individuais/Protocolo_de_condutas_perioperatorias/Protocolo_de_condutas_perioperatorias.pdf. 

## DISCUSSION

Abdominal wall hernias are common surgical conditions in the routine of the general surgeon, presenting a significant financial impact due to high hospital costs^33^ ^-^ ^36^. They vary in location and complexity, ranging from small umbilical hernias to complex recurrent hernias in patients with possible associated comorbidities^37^. The effectiveness of interventions may differ between populations, and the availability of resources may vary between hospitals, which requires specific perioperative protocols^38^. 

By identifying the perioperative procedures performed in a public health unit located in Northern Brazil to accelerate the postoperative recovery of patients undergoing abdominal wall surgeries, it was possible to show that the procedures undertaken by surgeons in this unit are in line with national and international guidelines on the matter. However, clinical-surgical approaches specifically related to abdominal wall surgeries are still incipient in the literature, thus reinforcing the importance of publishing the protocol derived from this study, aimed at health units in a Northern State capital. 

Protocols are essential to organize management and ensure patient safety^39^. Initiatives such as ERAS (Enhanced Recovery After Surgery) and ACERTO (Acceleration of Total Postoperative Recovery) promote modern protocols based on scientific evidence to optimize perioperative care and accelerate patient recovery, improving surgical outcomes and allowing earlier hospital discharges^17^ ^,^ ^36^ ^,^ ^40^ ^-^ ^43^.

However, the implementation of the ERAS (Enhanced Recovery After Surgery) protocol in abdominal wall hernia surgeries faces significant challenges due to preoperative, intraoperative, and postoperative factors that vary among patients, such as weight, nutritional status, and the presence of chronic diseases^19^ ^,^ ^44^. The success of hernioplasty depends to a large extent on the healing process, since the morbidity associated with the surgical wound can significantly influence the patient’s return to normal activity and quality of life^19^ ^,^ ^45^. 

Therefore, enhanced recovery programs that aim to mitigate the physiological damage caused by surgery have shown improvements in results and reduction in hospitalization time in gastrointestinal surgeries, also displaying benefits for abdominal hernias^15^ ^,^ ^19^ ^,^ ^38^ ^,^ ^46^. Nevertheless, there is a lack of evidence on the efficacy of these protocols specifically for abdominal hernias due to the heterogeneity of this surgical condition^15^ ^,^ ^19^ ^,^ ^46^ ^-^ ^48^.

In recent years, some studies have directly investigated the use of the ERAS protocol applied to the population with abdominal hernias^15^ ^,^ ^19^ ^,^ ^38^. Although they have indicated a decrease in the length of hospital stay, the efficacy of ERAS in complex abdominal wall hernias is still unknown^19^ ^,^ ^44^. Therefore, it is important for individual programs to identify specific interventions and opportunities for improvement in the conducts for this type of surgical pathology^38^. 

In this context, Orenstein et al.^49^ discussed complex abdominal wall reconstruction and ventral hernia repair, emphasizing the importance of optimizing patient comorbidities prior to surgery. The authors presented an enhanced recovery protocol to reduce complications and accelerate postoperative recovery^49^. On the other hand, Majumder et al.^50^ addressed the implementation of an enhanced recovery protocol for the repair of ventral hernias, emphasizing the importance of preoperative optimization and pain management^50^.

The protocol proposed in this study emphasizes the efficacy of perioperative practices in accelerating postoperative recovery in abdominal wall hernia surgeries, complementing the recommendations of the ERAS and ACERTO programs. We observed that weight loss was not considered a relevant factor for the risk of surgical wound infection in the hospital unit where we conducted the research. This finding contradicts the results of the systematic review by Marzoug, et al.^45^, which identified overweight as a critical factor for the risk of surgical wound infection. The divergence can be explained by the low incidence of obesity in the region where we conducted the study. Thus, we emphasize the importance of studies that assess standardized perioperative approaches adapted to regional specificities in Brazil.

The experts in this study highlighted the themes alcohol intake and tobacco use before the surgical procedure, referral to physical prehabilitation programs before major surgeries, and routine preoperative nutritional screening accompanied by nutritional supplementation and immunonutrition for malnourished patients. They also highlighted the non-routine use of nasogastric tubes or abdominal drains, the use of multimodal analgesia, and the use of risk scores for nausea or vomiting, along with the adoption of strategies for the prevention and treatment of these symptoms^15^ ^,^ ^19^ ^,^ ^51^. This shows that the conducts adopted by the professionals participating in this study are in line with what is currently being discussed about the acceleration of postoperative recovery in all phases of perioperative care. 

The informed consent form and the use of prophylaxis for venous thromboembolism, among others, did not reach a significant p-value at this stage, although they presented high degrees of agreement. The variation may have been caused by the interdependence of the questions, in which the answer to one was related to the information provided in the previous question. Also, individual clinical-surgical practices, developed from personal experience over the years, may have limited consideration of other important aspects of patients’ improved postoperative recovery. Finally, considering that the protocol is directed to a public health institution, some experts may have assumed that certain clinical-surgical practices would not be applicable in this context. 

The study achieved its goal by developing a protocol validated by local experts. This protocol integrates practices based on scientific evidence adapted to regional specificities, making it a useful tool for surgeons and multidisciplinary teams. The approach adopted resulted in an instrument that not only incorporates evidence-based best practices but also considers the particularities and resources available in the local health environment.

Although we followed all methodological rigor, some limitations associated with the study can be pointed out. Because the sample is composed of specialists chosen for convenience, there is the possibility of selection bias. In the first questionnaire, the following questions were applied from a text with the most current recommendations discussed on the topic, which may have generated a social desirability bias, as the participant may not have chosen the question that most pleased him/her because it was not in agreement with the current literature. 

Finally, there may be difficulties in the universal application of the results of this study, since it was conducted with experts familiar with local social, cultural, and financial characteristics, which may differ in other states or countries.

The strong point of achieving this consensus was the creation of a protocol of perioperative conducts for abdominal wall hernioplasties that will facilitate decision-making regarding the clinical-surgical conducts in the treatment of abdominal wall hernias, which will contribute to the safety and excellence of the surgical care provided. In addition, it will improve the clinical and surgical training of the new surgeons trained by the hospital unit where the study was carried out, advancing the health services offered by it.

## CONCLUSION

A perioperative management protocol, developed using the Delphi method with the contribution of specialists from a public health unit in Northern Brazil, can improve efficacy, efficiency, and safety of postoperative recovery in patients undergoing abdominal wall hernia surgery in the Public Unified Health System (SUS). The protocol considered regional specificities and the experiences of surgeons, allowing other health units in similar regions to use the material as support in decision-making.

The material produced is available for free at the link: https://www.poisson.com.br/livros/individuais/Protocolo_de_condutas_perioperatorias/Protocolo_de_condutas_perioperatorias.pdf
